# Frameworks for the design and reporting of anaesthesia interventions in perioperative clinical trials

**DOI:** 10.1016/j.bjao.2024.100374

**Published:** 2025-02-04

**Authors:** Karen D. Coulman, Lucy Elliott, Natalie S. Blencowe, Joyce Yeung, Leila Rooshenas, Robert J. Hinchliffe, Ronelle Mouton, collaborators, Eric Albrecht, David Bosanquet, Kariem El-Boghdadly, Gudrun Kunst, Mandeep Phull, Helen Galley, Ben Gibbison, Suzanne Harrogate, Anna Simpson, Jasmeet Soar, Michael Gillies, Mr James Glasbey, Sina Grape, David Hewson, Simon Howell, Louise Savic, Alan MacFarlane, Brendan McGrath, Ciara O'Donnell, Jon Silversides, Katrina Pirie, Andrew Toner

**Affiliations:** 9 Lausanne, Switzerland; 10 Cardiff, UK; 11 London, UK; 12 Aberdeen, UK; 13 Bristol, UK; 14 Edinburgh, UK; 15 Birmingham, UK; 16 Valais, Switzerland; 17 Nottingham, UK; 18 Leeds, UK; 19 Glasgow, UK; 20 Manchester, UK; 21 Belfast, UK; 22 Melbourne, Australia; 23 Perth, Australia; 1 Centre for Surgical Research, Population Health Sciences, Bristol Medical School, University of Bristol, Bristol, UK; 2 Translational Health Sciences, Bristol Medical School, University of Bristol, Bristol, UK; 3 Leeds Teaching Hospitals NHS Trust, Leeds, UK; 4 Warwick Clinical Trials Unit, Warwick Medical School, University of Warwick, Warwick, UK; 5 University Hospitals Birmingham NHS Foundation Trust, Birmingham, UK; 6 Population Health Sciences, Bristol Medical School, University of Bristol, Bristol, UK; 7 Bristol Surgery & Perioperative Care Complex Intervention Collaboration, Translational Health Sciences, Bristol Medical School, University of Bristol, Bristol, UK; 8 North Bristol NHS Trust, Bristol, UK

**Keywords:** anaesthesia interventions, protocol standards, randomised controlled trials, reporting standards

## Abstract

**Background:**

Interventions from RCTs can only be replicated and implemented if reported in sufficient detail. This study developed frameworks to assist researchers with describing, monitoring, and reporting the key components of anaesthetic interventions in trials.

**Methods:**

This study comprised three phases: (1) initial framework development—text describing the delivery of anaesthetic interventions was coded and categorised into components using thematic analysis; (2) refinement of frameworks—facilitated structured group discussions were conducted with perioperative clinicians, researchers, and journal editors to elicit additional framework categories and consider clarity and feasibility; (3) framework testing and further refinement—cognitive interviews with professionals undertaking trials evaluating anaesthesia interventions to test the feasibility of using the frameworks in contemporary perioperative trials.

**Results:**

Three frameworks were developed for general, regional, and sedation anaesthesia interventions. Data saturation of categories within the frameworks was reached after inclusion of 15 RCTs for general and regional anaesthesia, and 13 for sedation. Each framework is structured into three main sections: (1) professional(s) delivering the intervention; (2) setting; and (3) intervention components, with descriptions of the preoperative, intraoperative, and postoperative stages unique to each anaesthetic intervention. Each framework deconstructs an anaesthetic intervention into component parts to support researchers with the design and reporting of RCTs. Final frameworks are available at: https://anaesthesiaframeworks.blogs.bristol.ac.uk/.

**Conclusions:**

We provide novel frameworks to be used during the design of perioperative trials to facilitate the design, delivery, and reporting of anaesthesia interventions.

Perioperative care interventions such as anaesthesia are difficult to evaluate because they are complex, comprising multiple components each of which may to a lesser or greater degree impact on the overall clinical outcome.^1^ The complex nature of these interventions has major implications for the design and delivery of perioperative RCTs. The Medical Research Council (MRC) guidance for complex intervention development and evaluation and the Criteria for Reporting the Development and Evaluation of Complex Interventions in healthcare (CReDECI) provide some guidance on evaluating and reporting complex interventions, but these are not specific to RCTs or perioperative interventions.^1^^,^^2^ Guidance recommendations such as Template for Intervention Description and Replication (TIDieR), Standard Protocol Items: Recommendations for Interventional Trials (SPIRIT), Consolidated Standards of Reporting Trials (CONSORT), and CONSORT for non-pharmacological treatments (CONSORT-NPT) were published with the aim to improve the reporting of interventions in trials.^3^, ^4^, ^5^, ^6^ Although the above guidance recommendations are helpful, they are not always applicable to or specific enough for perioperative interventions. The reporting quality of perioperative interventions in trial protocols and reports has been found to be poor when evaluated against such reporting guidelines, and this problem is compounded by the acknowledged lack of consensus definitions and standardisation for anaesthetic interventions.^7^, ^8^, ^9^, ^10^, ^11^, ^12^, ^13^

It was shown that when surgical interventions are evaluated against the CONSORT-NPT guidelines, the precision with which they are described would permit replication in only about one-third.^14^, ^15^, ^16^ To address this, a framework was created to help with standardisation and monitoring of surgical interventions in trials, and this has been in use since 2016.^14^

A recent systematic review demonstrated significant variation in the descriptions of anaesthesia interventions, and protocol adherence was reported for only 12%.^17^ A lack of standardisation and consistency in how anaesthetic interventions are deﬁned, administered, and reported complicates the delivery and interpretation of perioperative trials. For example, the GALA trial compared general anaesthesia with local anaesthesia for patients undergoing carotid endarterectomy surgery in 3526 participants, but the inconclusive results were rendered questionable owing to the lack of details about the anaesthetic technique in both the control and intervention arms.^18^

The aim of this study was to develop frameworks for use at the design stage of perioperative trials to assist researchers in (1) describing the component parts of anaesthesia interventions within trial protocols, and (2) identifying which components need to be standardised and monitored within the particular trial context.

## Methods

**Figure 1:**
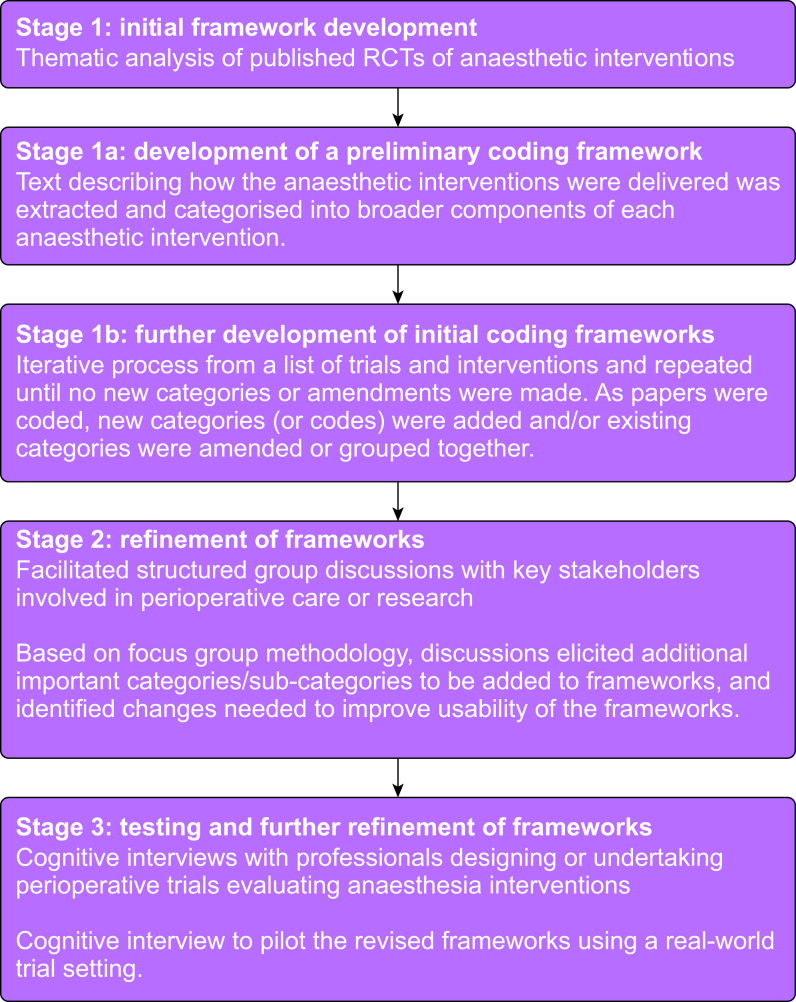
Summary of qualitative methodology to develop anaesthetic frameworks.

Qualitative methods were used across three research stages to develop and refine the frameworks (Fig 1). An individual framework was developed for each mode of anaesthesia: (1) general anaesthesia (GA), (2) regional anaesthesia (RA), and (3) sedation. The frameworks were informed by a recent systematic review of how anaesthesia interventions are described, standardised, and monitored in published RCTs.^17^ The aim was to ensure that the frameworks were comprehensive and detailed enough in terms of categories and descriptors to support a full description of anaesthetic interventions within trial protocols, while ensuring the frameworks were presented clearly and logically.

### Stage 1: initial framework development: thematic analysis of published RCTs of anaesthetic interventions

Thematic analysis of qualitative data was used to develop the initial frameworks as previously described and used to develop a framework for standardising surgical interventions in RCTs^14^ (Fig 1).

#### Stage 1a: development of preliminary coding frameworks

Preliminary coding frameworks were developed for GA, local anaesthesia, and sedation using six exemplar anaesthesia trial reports and associated protocols, where available.^18^, ^19^, ^20^, ^21^, ^22^, ^23^, ^24^, ^25^, ^26^ These exemplar papers were selected based on expert clinical knowledge of the area before undertaking our systematic review (see stage 1b). Papers were scrutinised for text describing how the anaesthetic interventions were delivered. Relevant text was extracted and categorised into broader components of each anaesthetic intervention. This was undertaken independently by five of the study authors (KC, LE, NB, RH, RM) who then met to compare and discuss their categorisations. A preliminary framework for each mode of anaesthesia was drawn up by LE and KC following this meeting; these were used as coding frameworks for stage 1b.

#### Stage 1b: further development of the initial frameworks

Trial reports identified in our systematic review assessing the reporting quality of anaesthetic interventions in RCTs formed the basis of the data used to further develop the initial frameworks.^17^ The review identified 162 anaesthetic interventions across 78 RCTs. Studies were purposefully selected from the systematic review to ensure a range of foci and interventions were represented in the resulting frameworks. We selected papers published in anaesthetic journals (rather than medical or surgical journals) because of an increased likelihood of detailed descriptions of anaesthetic interventions, which would contribute more data. The selection of papers for further development of the frameworks was iterative and was reviewed throughout the coding process. The Methods sections of included papers and protocols were coded by KC using the preliminary coding frameworks developed in stage 1a. Coding and data management was facilitated using NVivo (release 1.7.1). As papers were coded, new categories (or codes) were added, existing categories were amended/grouped together, or both, where appropriate. These evolving coding frameworks were reviewed regularly with all study authors (after each batch of five papers had been coded) where further amendments were made as required. Decisions about the selection of further papers to include in the coding process were also decided upon in these meetings. Previously coded trial reports were re-coded as appropriate as the framework was updated. This iterative process was repeated until ‘data saturation’ had been reached for each mode of anaesthesia—the point at which the research team felt that no new categories or amendments were being made to the frameworks with the inclusion of additional papers.^27^

### Stage 2: refinement of frameworks: facilitated structured group discussions with key stakeholders involved in perioperative care or research

A key informant approach to purposive sampling was used to invite UK and international experts in perioperative research to participate in facilitated structured group discussions, based on focus group methodology.^28^^,^^29^ These group discussions were used to elicit additional important categories/sub-categories to be added to the frameworks, and identify any changes needed related to wording and ordering to improve usability of the frameworks (see Supplementary Appendix S1 for group discussion topic guide). Participants consisted of researchers from both surgery and anaesthesia specialties involved in perioperative clinical trials and editors of journals relevant to perioperative medicine trials. Key informants were initially approached through professional networks such as the Perioperative Medicine Clinical Trials Network (POMCTN) and the Anaesthesia Research Society (ARS); this was followed by snowball sampling, with initial key informants suggesting further key informants to invite to the research.

Detailed notes of group discussions were taken by KC or LE. Group discussions were recorded and transcribed to cross-reference with meeting notes. After completion of all group discussions, a rapid thematic analysis of meeting notes was undertaken to identify key themes relating to suggested additions and changes to the frameworks. Suggested changes were discussed and agreed at a full study meeting, and subsequently incorporated into the frameworks before stage 3.

### Stage 3: testing and further refinement of frameworks: cognitive interviews with professionals who are leading perioperative trials evaluating anaesthesia interventions

International healthcare professionals undertaking perioperative trials evaluating anaesthesia were invited to take part in a cognitive interview to pilot the revised frameworks using a real-world trial setting.^30^ Participants reviewed the framework(s) most relevant to their trial and were asked to think specifically about how the framework would apply to their trial throughout the interview, using a ‘think aloud’ approach.^31^ Participants were also asked to provide suggestions on how intervention standardisation and monitoring could practically be embedded within the frameworks, and the best format for accessing the frameworks (see Supplementary Appendix S2 for interview topic guide). Interviews were recorded and transcribed.

Interview transcripts were analysed to identify ‘response problems’ to individual items on the framework based on a standardised classification scheme as described by Horwood and colleagues,^32^ which included problems with (1) comprehension or clarity; (2) comprehensiveness; (3) interpretation; and (4) logic of item ordering/ease of navigation. A thematic analysis of transcripts was also undertaken to provide additional insights about possible improvements to the frameworks.^33^ NVivo software was used to aid with management of qualitative data. Analysis provided detailed information on revisions needed to the framework.

### Patient and public involvement

The Royal College of Anaesthetists Patient Carer Public Involvement Engagement (PCPIE) group reviewed and commented on the frameworks at the preliminary development stage. They commented that just as ‘consistent use of terminology and information will help researchers to make accurate comparisons between studies, for patients the consistency of reporting of interventions should lead to a consistency and predictability of the delivery of anaesthesia and patients need a greater understanding of the appropriateness of the anaesthesia to their physical and mental situation’. It was also acknowledged that patients being asked to participate in a focus group, looking at the different stages of anaesthesia, felt inappropriate, given that ‘patients are unconscious or unaware for much of the procedure, particularly if they are given a general anaesthetic’.

## Results

### Papers included in framework development (stage 1)

**Figure 2:**
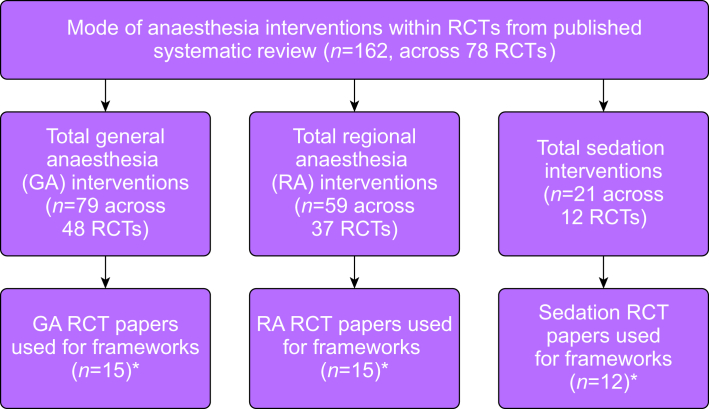
Identification of research publications to develop anaesthetic frameworks. ∗In total, these represented 39 distinct RCTs: 13 reported GA interventions only, 13 RA interventions only, 10 sedation interventions only, one reported GA and RA interventions, one reported GA and sedation interventions, and one reported RA and sedation interventions.

Out of the 78 RCTs identified in our systematic review, 39 were purposefully selected to develop the three initial frameworks. Of these RCTs, 15 reported GA interventions, 15 RA interventions, and 12 sedation interventions (Fig 2). Four associated study protocols (two GA, one RA, one sedation) were also obtained. The characteristics of RCTs used to develop each framework are described in Supplementary Tables S1–S3 with references in Supplementary Appendix S3.

### Facilitated structured discussions and cognitive interview participants (stages 2 and 3)

In total, 22 participants took part in steps 2 and 3 which included three facilitated structured group discussions and nine individual interviews (13 participants took part in a group discussion, seven in an interview, two in both). This included 20 anaesthetists and two surgeons, 17 of whom also held academic roles and eight of whom also held journal editor roles. Each group discussion had four to six participants and lasted 2 h. Interviews lasted for 33–75 min. A summary of key changes made to the initial frameworks after the group discussions and interviews is outlined in Supplementary Appendix S4.

### The frameworks

The full final frameworks for GA, RA, and sedation interventions can be accessed here: https://anaesthesiaframeworks.blogs.bristol.ac.uk/. Each framework is separated into three sections: professional delivering the anaesthetic intervention, setting of the anaesthetic intervention, and intervention components (Table 1). All three frameworks include a description of the expertise of the professional delivering the anaesthetic, to include grade and specialty, and whether a required expertise has been stipulated by the study team. The setting or location in which the procedure is to be performed includes whether the intervention is performed in a hospital, non-hospital setting, or on a transfer, and if in a hospital, the specific location.

**Table 1:** Main heading structure of the frameworks. Please see https://anaesthesiaframeworks.blogs.bristol.ac.uk/for complete frameworks with all components.

1) Mode of anaesthesia (general, regional, or sedation)
*Regional specific: type and location: peripheral nerve block, neuraxial, infiltration*
2) Professional delivering anaesthetic intervention
a) Specialty
b) Grade
c) Required expertise in delivering study intervention (study team to define)
3) Setting where anaesthetic is being delivered
a) Hospital location
b) Non-hospital location
4) Intervention components (see Table 2 for details for each intervention)
a) Preprocedural medications (additional to patient's usual medications)
i) Drug name, dose, route, timing and reason for administration, timing
ii) *Regional specific: patient positioning, state of consciousness, consent and correct site identification, preparation of sterile field*
b) General anaesthesia/regional anaesthesia/procedural sedation
c) Recovery from anaesthesia
i) Medication given postprocedure, additional i.v. fluids, monitoring, initial and subsequent postprocedure destinations

The ‘intervention components’ section includes three stages of an anaesthetic intervention; components that take place before surgery, during the anaesthetic intervention, and in the recovery from anaesthesia. Preoperative components include details of any preprocedural medications used, included name, dose, route of administrations, timing of administration, and the reason.

For procedural components, all three frameworks include a description of monitoring used and whether this was standard monitoring stipulated by that study centre, or any additional monitoring. Each framework includes a description of any anaesthetic agents administered during the procedure, to include dose and route for each, and whether further medication was administered during the procedure in addition to initial medications as part of the anaesthetic procedure.

All three frameworks have the same ‘recovery from anaesthesia’ descriptors to include details of the initial postprocedure destination and the subsequent destination of the patient, a description of all monitoring used, and any medication administered for recovery of the patient, to include name, dose, timing, route, and reason for administration. The main intervention components that take place during the intervention are described in Table 2 for each mode of anaesthesia.

**Table 2:** Main intervention components within the frameworks for each mode of anaesthesia.

General anaesthesia (GA)	Regional anaesthesia (RA)	Sedation
**Monitoring** Standard Additional Frequency	**Technique** Landmark Neurostimulator Ultrasound guidance	**Patient positioning**
**Supplementary oxygen** Nasal cannulae Mask Flow rate Duration	**Needle details** Type or make Size/diameter (G) Length Single shot (Y or N) Catheter (Y or N)	**Monitoring** ECG Oxygen saturations Noninvasive blood pressure ETCO_2_ Temperature Processed EEG Respiratory rate Other Frequency of monitoring
**I.V. fluids** Type of fluid Volume or rate	**Local anaesthetic to skin** Drug name Drug dose	**Supplementary oxygen** Nasal cannulae Mask Flow rate Duration
**Airway management** Endotracheal tube Supraglottic device Mask Tracheostomy Other (specify) Ventilator settings	**Regional drug details** Test dose (Y or N) Drug name Drug dose Timing of administration	**I.V. fluids** Type of fluid Volume or rate
**Drugs given at induction** Drug name, dose, route and time of administration	**Administration of regional injection (Y or N to all)** Easy to inject Negative aspiration Good spread of injectate	**Sedation drugs used** Bolus (Y or N) Infusion (Y or N) Other modes of sedation (details)
**Maintenance of GA** Total intravenous anaesthesia (TIVA) Volatile	**Assessment of block (if applicable)** Sensory scale Motor scale Frequency of assessment Definition of successful block Time to onset of block Duration of block Other	**Variables** **used for sedation titration** Depth of sedation Pain Other physiological variables (describe)
**Drugs given at emergence** Drug name, dose, route and time of administration	**Escalation of care (if required)** Regional block or additional regional block Local anaesthetic infiltration Sedation Additional analgesia Other additional agents General anaesthesia Reason for additional intervention	**Additional drugs given** Drug name, dose, route, timing, and reasons for administration
**Extubation details**	**Notable events during regional procedure** Needle passes Failed attempts Pain on injection Bleeding at puncture site Paraesthesia during block procedure	**Unplanned additional techniques** Local anaesthetic infiltration Regional anaesthesia General anaesthesia Airway manoeuvre or assistance Other Reason for additional intervention
	**Complications of regional procedure** Immediate Longer-term	
	**Patient positioning after regional procedure** Supine Lateral Sat up Prone Other (specify)	

The frameworks offer specific descriptions of the procedural stage of the intervention components unique for each mode of anaesthesia. For the GA framework, additional descriptors are provided for airway management, drugs given for induction and maintenance of anaesthesia as well as mode of anaesthesia maintenance, and emergence and extubation details. For the RA framework, a description of preprocedural preparation is included, followed by detailed description of the exact technique including details of drugs and equipment used, assessment of the regional block, and any additional aspects associated with the technique, including complications or any requirement for escalation of care or deviation from the initial regional anaesthetic plan. The sedation framework includes the mode of delivery of sedation drugs used, parameters used for sedation titration, and whether unplanned additional techniques were required and for what reasons.

## Discussion

In response to the challenge of poorly reported anaesthesia interventional studies, we have developed three novel frameworks for use in the design and reporting of GA, RA, and sedation clinical trials. These frameworks provide a means to deconstruct each anaesthetic intervention into their component parts, to ensure that the standardisation of each component is considered *a priori,* benefitting researchers in the planning and reporting of anaesthetic clinical trials. We recognise that not all aspects of the frameworks will be applicable for each trial and therefore we are not mandating every component of the frameworks be collected, standardised, and reported for every trial. The frameworks can support researchers in considering each component part of their anaesthetic intervention and deciding which need to be standardised and monitored for their particular trial.

We are not the first study to recognise that tools to improve the standardisation of anaesthetic interventions are needed. Recognition of the heterogeneity in nomenclature of regional anaesthetic techniques resulted in the first international consensus on descriptions of regional techniques of the abdominal wall, chest wall, and paraspinal blocks, with recommendations for use both in research and clinically.^34^ More recently, a modified Delphi survey has been performed to establish expert consensus on documentation for regional anaesthetic interventions in clinical practice, following recognition of the lack of recommendations for key documentation for such anaesthetic interventions.^35^ The authors suggested that the compiled list from their study might provide a framework to guide documentation standards in the future. Work has been done recently to provide a specific definition for procedural sedation after it was identified that significant ambiguity with the definitions of sedation was leading to incomplete and inconsistent reporting of procedural sedation.^36^ This same group also looked at the reporting of sedation-related outcomes and demonstrated how this area of reporting is subject to substantial variability.^37^ They have subsequently developed a tool with the aim to reduce reporting variation for sedation interventions. This tool focuses on reporting sedation outcomes, whereas our frameworks focus on how to describe the delivery of the intervention.

### Use of the frameworks to design an RCT

At the outset of a trial, we suggest the frameworks are used by perioperative triallists to help develop the trial protocol. For example, the triallists may decide that some components of the anaesthesia intervention are mandatory (e.g. the setting: it must be done in a day-case operating theatre), whereas other aspects of the intervention are not mandatory (e.g. it is not important what grade of anaesthetist gives the anaesthetic). The frameworks do not capture all aspects of contemporary anaesthesia practice, and we recognise that different modes of anaesthesia can be used as an adjunct to the main trial intervention. For example, if the main intervention being studied is a form of RA, the framework can be used to describe which components of the RA intervention are mandatory, optional, or prohibited, whilst it is up to trialists to decide to what extent they stipulate adjuncts, which in this case can be GA, sedation, or additional local anaesthesia. It is important that the team members are explicit about all the chosen components of the framework to be studied in the protocol such that the intervention can be replicated, and the trial has external validity. There is a spectrum of acceptable variation over which adherence to a protocol could be considered, and this will be specific to each trial. The frameworks therefore provide an aid to trialists to determine how they wish to monitor adherence to the specific aspects of the intervention in their trial.

### Use of the frameworks to report and assess intervention fidelity

It is important to report how well the trial adhered to the key components of interventions. In the example above, it would be a protocol violation if some centres did not perform the anaesthesia in a day-case operating theatre, and this would impact on the interpretation of the trial. The extent to which each component of perioperative interventions is standardised depends on whether a trial is predominantly pragmatic or explanatory. Pragmatic trials assess whether interventions are effective in the real world and often involve multiple centres and healthcare professionals. To achieve generalisation, a balance is needed between practicality and adequate standardisation. In contrast, exploratory trials test the efficacy of interventions under optimal conditions and may require more detail to assess the safety of interventions, which are often novel.^14^^,^^38^ Consequently, the frameworks need to be adaptable to encompass the spectrum of RCT settings. In a pragmatic trial therefore, after identifying all components, the ‘key’ components can be agreed and standardised, and remaining components delivered flexibly according to the trial team. To illustrate: the NIHR-HTA-funded CAMELOT trial evaluates the efficacy of rectus sheath catheter analgesia in people who have emergency laparotomy surgery with a midline incision (ISRCTN 15475290).^39^ To support standardisation and adherence, it is mandated that the rectus sheath catheters are inserted in the operating theatre, before closure of the laparotomy wound, by a surgeon who received trial-specific training of the mandatory, optional, and prohibited components of rectus sheath catheter insertion. The subsequent local anaesthesia infusion via the surgically inserted rectus sheath catheters via a pump is not mandated and sites may choose from a recommended list of equipotent alternatives in routine clinical use for nerve block catheter. This will ensure the trial remains pragmatic and reflects current practice.^40^

The issue of standardising the reporting of complex interventions is not unique to anaesthesia. Assessment of the reporting of surgical interventions against the CONSORT-NPT guidelines has shown that poor compliance of intervention reporting creates difficulties in how results can be evaluated.^41^ Subsequently, a reporting framework was created to improve the reporting of surgical interventions in trials.^14^^,^^15^ Attempts have also been made to develop reporting standards for other areas of medicine, such as clinical studies in vascular surgery and diabetes, but these were developed largely by expert consensus and were therefore methodologically limited and were not specific to RCTs.^42^^,^^43^

We recognise that the process of developing these frameworks is not without bias. The frameworks are deliberately simplistic to ensure that all options of each mode of anaesthesia could be included and this may present as a ‘right’ or ‘wrong’ way to perform an intervention, whereas it is at the discretion of the research team as to which aspects of the frameworks are relevant for use for their intervention to be studied. Additionally, using trials selected from high-impact journals reported in our systematic review is likely to have limited what has been included in the frameworks and may mean that the frameworks do not include all components felt to be relevant to each anaesthetic intervention. Likewise, although this process has been successfully applied to surgical interventions, this process has not previously been used in anaesthetic interventions and therefore it is unknown how transferable the methodology is. To mitigate for this, the draft frameworks underwent testing through group discussions and cognitive interviews with perioperative experts. Throughout the development of the frameworks, there has been engagement and support from key stakeholder groups.

We believe that developing frameworks for anaesthesia will aid research teams in describing, standardising, and monitoring aspects of these complex interventions in perioperative clinical trials. This should improve the translation of evidence-based research into day-to-day clinical practice. These frameworks are not static and can be adapted as anaesthesia interventions evolve. Future steps for our project will focus on wider validation with a wider group of international professions and a more multidisciplinary group. Finally, we intend to promote the use of the frameworks by those designing, delivering, and reporting RCTs which should ensure further stakeholder buy-in prior to wider dissemination into the research community.

### Author contributions

KDC: Study design, participant recruitment, data collection and analysis, writing up of the first draft of the paper; LE: Participant recruitment, data collection and analysis, writing up of the first draft of the paper; NSB: Study conception and design, data interpretation, commenting on drafts of the paper; JY: Study design, participant recruitment, data interpretation, commenting on drafts of the paper; LR: Study design, data collection, data interpretation, commenting on drafts of the paper; RJH: Study conception and design, data interpretation, commenting on drafts of the paper; RM: Study conception and design, participant recruitment, data collection, data interpretation, commenting on drafts of the paper.

## Funding

Association of Anaesthetists and National Institute of Academic Anaesthesia; National Institute for Health and Care Research Bristol Biomedical Research Centre; NSB is an MRC Clinician Scientist. Health Education England (HEE)/National Institute for Health Research (NIHR) Clinical Lectureship (ICA-CL-2018-04-ST2-008 to KDC); The Royal College of Surgeons Bristol Centre for Surgical Research at the University of Bristol (to KDC); Linder Foundation & Royal College of Surgeons of England (to RJH and NB); North Bristol Vascular Charitable Fund (RM). The views expressed are those of the author(s) and not necessarily those of the MRC, NIHR, or the Department of Health and Social Care.

## Declaration of interest

The authors declare that they have no conflicts of interest.

## Appendix A List of collaborators

Professor Eric Albrecht, Lausanne, Switzerland

Mr David Bosanquet, Cardiff, UK

Professor Kariem El-Boghdadly, London, UK

Professor Helen Galley, Aberdeen, UK

Dr Ben Gibbison, Bristol, UK

Professor Michael Gillies, Edinburgh, UK

Mr James Glasbey, Birmingham, UK

Dr Sina Grape, Valais, Switzerland

Dr Suzanne Harrogate, Bristol, UK

Dr David Hewson, Nottingham, UK

Dr Simon Howell, Leeds, UK

Professor Gudrun Kunst, London, UK

Professor Alan Macfarlane, Glasgow, UK

Professor Brendan McGrath, Manchester, UK

Dr Ciara O’Donnell, Belfast, UK

Dr Mandeep Phull, London, UK

Dr Katrina Pirie, Melbourne, Australia

Dr Louise Savic, Leeds, UK

Dr Jon Silversides, Belfast, UK

Dr Anna Simpson, Bristol, UK

Dr Jasmeet Soar, Bristol, UK

Dr Andrew Toner, Perth, Australia
