# Supplementary material for: Frameworks for the design and reporting of anaesthesia interventions in perioperative clinical trials
Source: BJA Open. 2025 Feb 4;13:100374. doi: 10.1016/j.bjao.2024.100374 (PMC11847521; doi:10.1016/j.bjao.2024.100374)
Supplement: Multimedia component 1 [file mmc1.docx]

**Supplementary Appendix S1: Facilitated structured group discussion topic guide**

**Standardised frameworks to improve the reporting quality of anaesthesia interventions in clinical trials**

NB This guide is necessarily provisional, as its application will depend on the experience of individual participants.

1. Introductions and background information
   1. Welcome to the meeting from Chair
   2. Brief Introductions - attendees
   3. Explaining the aims and objectives of the study and work undertaken to date
   4. Plans for the group discussion today – how it will work and any ground rules
2. General anaesthesia framework
   - *To go through section by section, starting with higher level headings first*
     - Pre-operative medications
       - Are any subclasses of medications needed here?
     - General anaesthesia
       - Some components are capitalized to represent the main components of GA. Do we like this or is it confusing? Should we make all lower-case?
       - ‘Intra-operative analgesia’ – is timing relevant here? Is ‘timing’ (as per TIDIER) relevant to other categories too?
       - ‘Extubation details’ – are sub-categories needed?
     - Recovery from anaesthesia
       - ‘Monitoring in Recovery’ – are sub-categories needed?
   - Questions to ask of each section:
     - Are any components missing from this section?
     - Are components worded appropriately/are changes needed to the wording?
     - Are intervention components ordered appropriately?
     - How practical/easy is it to describe these components in a consistent manner? Could it be consistently used across many studies.
3. Regional anaesthesia framework
   - *To go through section by section, starting with higher level headings first. ‘Recovery from anaesthesia’ section may not need to be reviewed again as the same for GA.*
     - Pre-regional anaesthesia preparation
     - Regional anaesthesia
     - Recovery from anaesthesia
   - Questions to ask of each section:
     - Is anything missing from this section?
     - Is it worded appropriately/are changes needed to the wording?
     - Are intervention components ordered appropriately?
     - How practical/easy is it to describe these elements in a consistent manner? Could it be consistently used across many studies.
4. Sedation anaesthesia framework
   - *To go through section by section, starting with higher level headings first. ‘Pre-operative medications’ and ‘Recovery from anaesthesia’ section may not need to be reviewed again as the same for GA.*
     - Pre-operative medications
       - Are any subclasses of medications needed here?
     - Procedural Sedation
     - Recovery from anaesthesia
   - Questions to ask of each section:
     - Is anything missing from this section?
     - Is it worded appropriately/are changes needed to the wording?
     - Are intervention components ordered appropriately?
     - How practical/easy is it to describe these elements in a consistent manner? Could it be consistently used across many studies.
5. [IF DOESN’T COME UP EARLIER]: Should each medication throughout the typologies include the following sub-components: i) agent, ii) dose, and iii) route (as for the pre-operative medications)? E.g. ‘Induction agents including dose’. Or if that makes it too clunky, perhaps somewhere at the beginning it should be specified that there are certain features of a medicine (i.e. in line with TIDIER) that should be considered and give the list once for the purposes of illustration?
   1. *Karen can explain this more if needed*
6. ‘Expertise’ section of the framework (applies to all three)
   - Is anything missing from this section?
     - Is it possible that this would be different for different parts of the intervention, and do we need to incorporate this?
   - Is it worded appropriately/are changes needed to the wording?
   - How practical/easy is it to describe these elements in a consistent manner? /Could it be consistently used across many studies.
7. ‘Setting’ section of the framework (applies to all three)
   - Is anything missing from this section?
     - Does this need to be separated out for different aspects of the intervention, e.g. pre-anaesthesia, anaesthesia and post-anaesthesia/recovery?
   - Is it worded appropriately/are changes needed to the wording?
   - How practical/easy is it to describe these elements in a consistent manner? /Could it be consistently used across many studies.
8. [IF DOESN’T COME UP EARLIER]: How do people feel about the overall structure (three main headings)?
9. Overall feasibility considerations
   1. How can we prevent framework fatigue?
   2. Should we aim to incorporate this framework into CONSORT or other frameworks??
   3. How would we best ‘advertise’ the frameworks/get buy-in/ensure they are used?
   4. *Other feasibility considerations?*
10. Other thoughts or issues
11. Close of meeting
    1. Thank you
    2. Re-cap what will happen to these data and next stages
