# Supplementary material for: Frameworks for the design and reporting of anaesthesia interventions in perioperative clinical trials
Source: BJA Open. 2025 Feb 4;13:100374. doi: 10.1016/j.bjao.2024.100374 (PMC11847521; doi:10.1016/j.bjao.2024.100374)
Supplement: Multimedia component 2 [file mmc2.docx]

**Supplementary Appendix S2: Interview topic guide**

**Standardised frameworks to improve the design of anaesthesia interventions in clinical trials**

NB This guide is necessarily provisional, as its application will depend on the experience of individual participants.

**Pre-interview:**

- *Brief introduction to background and rationale for study to develop frameworks for anaesthetic interventions*
- *Brief summary of work undertaken so far to develop and update frameworks (three frameworks developed) including literature review and focus groups.*
- Now we are asking professionals who are currently designing or undertaking perioperative trials evaluating anaesthesia interventions to test out these frameworks so we can understand how they work in a real-world trial situation. You have been invited to participate because you are involved in the [NAME] trial.
- The purpose of today’s interview is to discuss/pilot/pre-test the [GA/RA/sedation – *as applicable to the participant’s trial*] framework to get feedback on the content and the structure, and how this would best work in a real-world trial situation.
- *Check potential participant has read and understood the information sheet and ask if they have any questions so far*
- We’ll start off by running through the consent form – I’ll switch the audio-recorder on and take verbal consent to ensure you are happy to proceed. We will then go on to the main interview.
- Are you happy for the audio-recorder to be switched on?
- *Read through statements on consent form and ask participant to agree/decline each statement accordingly.*
- *If consent obtained, confirm that will now proceed with interview. Consent form to be emailed to participant after. Remind participant that they are free to end interview at any time.*

**Main Interview**

**Introduction and trial context**

- Could you start off by describing your professional role?
  - Tell me about your experience working in anaesthesia trials?
- Could you tell me about the [NAME]/anaesthetic trial you are in the process of designing/conducting? (*If there is more than one, ask them which they would like to think through today when going through the framework*)
  - Could you describe the trial intervention and comparator?

**Discussion of the framework relevant to their trial**

*Briefly present the three/four main sections of the framework. (Participant should have had link to frameworks sent in advance so can either have it up on their computer or researcher can also share screen*).

- What are your views on the definition of anaesthesia used in the framework as applicable to your trial?
- [IF APPLICABLE – For RA TRIALS ONLY] Reading through the **‘Type of RA’** section:
  - Do the categories and items in this section make sense to you?
    - Do you understand what they mean?
    - Are there any words that are confusing?
  - Are you able to accurately describe the type of RA in your trial from the categories and items included? **First thinking about the intervention and then the comparator *(if applies to both*).**
    - Is anything missing?
  - How clear/easy is this section to navigate?
    - What do you think about the general order of the categories and sections and how these are organized?
    - Are there parts that could be clearer?
- Reading through the **‘Operator’** section
  - Do the categories and items in this section make sense to you?
    - Do you understand what they mean?
    - Are there any words that are confusing?
  - Are you able to adequately describe the operator of the anaesthetic intervention in your trial from the categories and items included? **First thinking about the intervention and then then the comparator.**
    - Is anything missing?
  - How clear/easy is this section to navigate?
    - What do you think about the general order of the categories and sections and how these are organized?
    - Are there parts that could be clearer?
- Reading through the **‘Setting’** section
  - Do the categories and items in this section make sense to you?
    - Do you understand what they mean?
    - Are there any words that are confusing?
  - Are you able to adequately describe the setting of the anaesthetic intervention in your trial from the categories and items included? **First thinking about the intervention and then then the comparator.**
    - Is anything missing?
  - How clear/easy is this section to navigate?
    - What do you think about the general order of the categories and sections and how these are organized?
    - Are there parts that could be clearer?

**Now moving onto the Intervention Components section which is the most detailed**

- Reading through the **‘Pre-operative anaesthetic medications/Pre-regional anaesthesia preparation’** (as applicable) section
  - Do the categories and items in this section make sense to you?
    - Do you understand what they mean?
    - Are there any words that are confusing?
  - Are you able to fully describe the ‘pre-operative anaesthetic medications/pre-regional anaesthesia preparation’ in your trial from the categories and items included? **First thinking about the intervention and then then the comparator.**
    - Is anything missing?
  - How clear/easy is this section to navigate?
    - What do you think about the general order of the categories and sections and how these are organized?
    - Are there parts that could be clearer?
- Reading through the **‘GA/RA/Procedural Sedation’** (as applicable) section
  - Do the categories and items in this section make sense to you?
    - Do you understand what they mean?
    - Are there any words that are confusing?
  - Are you able to fully describe the GA/RA/Procedural sedation in your trial from the categories and items included? **First thinking about the intervention and then then the comparator.**
    - Is anything missing?
  - How clear/easy is this section to navigate?
    - What do you think about the general order of the categories and sections and how these are organized?
    - Are there parts that could be clearer?
- Reading through the **‘Recovery from anaesthesia/procedural sedation’** (as applicable) section
  - Do the categories and items in this section make sense to you?
    - Do you understand what they mean?
    - Are there any words that are confusing?
  - Are you able to fully describe the recovery from anaesthesia/procedural sedation in your trial from the categories and items included? **First thinking about the intervention and then then the comparator.**
    - Is anything missing?
  - How clear/easy is this section to navigate?
    - What do you think about the general order of the categories and sections and how these are organized?
    - Are there parts that could be clearer?

**Intervention standardization and monitoring**

Thank you for thinking through all of that. Another area that we would like to build into these frameworks is how they could help trialists decide when and how individual components of the interventions should be standardized and monitored within a trial.

So first they would help the triallists to fully describe all the different components of their interventions within the trial protocol (everything we have just been through), and then from this detailed description, triallists would decide which of these components need to be standardized and monitored within their particular trial.

We haven’t currently included any sections or items related to standardization or monitoring within the frameworks.

- Would you find it helpful for these elements to be included?
- How might we embed these elements within the frameworks in a user-friendly way?

**Overall including Format/layout**

- Is there anything else that is missing from the frameworks that you haven’t already mentioned?
- How do you find the overall format/layout of the frameworks?
- What would be the best format/layout for accessing the frameworks?
- Any suggestions for how it could be designed differently?

**Overall utility**

- Would this framework be helpful to you in designing, conducting and reporting an anaesthesia trial?
- How likely would you be to use it at:
  - Design stage? (E.g Helping you to think through all the important elements and which should be standardized?
  - During trial conduct (monitoring)
  - During trial reporting?
- How could it be more helpful?

**Wrap-up**

- Thank you for your time
- *Ask for any known colleagues/names that might be suitable to interview*
