# Supplementary material for: Frameworks for the design and reporting of anaesthesia interventions in perioperative clinical trials
Source: BJA Open. 2025 Feb 4;13:100374. doi: 10.1016/j.bjao.2024.100374 (PMC11847521; doi:10.1016/j.bjao.2024.100374)
Supplement: Multimedia component 3 [file mmc3.docx]

**Supplementary Appendix S3: Supplementary Tables S1-S3**

**Supplementary Table S1: Randomised controlled trial papers used to develop General Anaesthesia framework (n=15)**

| **Randomised controlled trial** | **Study details *(details used in purposive sampling, e.g. interventions compared, clinical area)*** |
| --- | --- |
| Abrons *et al*, 2017(1) | Conventional versus bougie technique nasotracheal intubation in GA, clinical area(s) not specified |
| Basciani *et al*, 2016(2) | Etomidate versus propofol versus etomidate + hydrocortisone in GA, cardiothoracic surgery |
| Beck-Schimmer *et al*, 2016(3) | Intravenous propofol versus volatile desflurane in GA, lung surgery |
| Dutta *et al*, 2019(4) | Propofol + dexmedetomidine versus no dexmedetomidine in GA, upper GI surgery |
| Ammar *et al*, 2016(5) | Propofol versus isoflurane in GA, cardiothoracic surgery |
| Gebhardt *et al*, 2018(6) | GA versus spinal anaesthesia, orthopaedic surgery |
| Mayr *et al*, 2016(7) | GA versus sedation, cardiothoracic surgery |
| Mu *et al*, 2018(8) | Target-controlled infusion (TCI) versus manual infusion in GA, paediatric surgery |
| Oh *et al*, 2018(9) | Propofol versus sevoflurane in GA, breast surgery |
| Ryu *et al*, 2018(10) | Desflurane versus sevoflurane versus in GA, orthopaedic surgery |
| Koo *et al*, 2018(11) | Moderate versus deep neuromuscular blockade, colorectal surgery |
| YaDeau *et al*, 2018(12) (+ YaDeau protocol, 2018(13)) | GA versus spinal anaesthesia as addition to popliteal and adductor canal nerve blocks, orthopaedic surgery |
| Zhang *et al*, 2018(14) (+ Zhang protocol(15)) | Propofol versus sevoflurane in GA, cancer surgery |
| Choi *et al*, 2019(16) | Rocuronium bolus versus continuous infusion in GA, paediatric surgery, |
| Fuchs-Buder *et al*, 2019(17) | Deep versus moderate neuromuscular block in GA, upper GI surgery |

**Supplementary Table S2: Randomised controlled trial papers used to develop Regional Anaesthesia framework (n=15)**

| **Randomised controlled trial** | **Study details *(Interventions compared, clinical area)*** |
| --- | --- |
| Aitken *et al*, 2016(18) (+ Macfarlane *et al*, 2013 protocol(19)) | Regional brachial plexus block versus local anaesthesia, arteriovenous fistula formation |
| Arnuntasukapul *et al*, 2018(20) | Lumbar plexus block - ultrasound with neurostimulation versus ultrasound alone, orthopaedic surgery |
| Boivin *et al*, 2016(21) | Axillary versus infraclavicular block, orthopaedic surgery |
| Bong *et al*, 2019(22) | Dexmedetomidine sedation with caudal block versus GA, paediatric surgery |
| Gebhardt *et al*, 2018(6) | GA versus spinal anaesthesia, orthopaedic surgery |
| Grape *et al*, 2019(23) | Supraclavicular versus retroclavicular brachial plexus block, orthopaedic surgery |
| Chin *et al,* 2018(24) | Neuraxial ultrasound versus standard scan in combined spinal-epidural, obstetrics |
| Li *et al*, 2019(25) | Ultrasound examinations versus landmark technique in spinal anaesthesia, obstetrics |
| Ryu *et al*, 2018(26) | Ultrasound assisted versus fluoroscopic-guided lumbar sympathetic ganglion block, pain medicine |
| Echaniz *et al*, 2019(27) | Bilateral suprazygomatic maxillary nerve blocks versus infraorbital and palatine nerve blocks, cleft palate surgery |
| Auyong *et al*, 2017(28) | Ultrasound-assisted versus palpation thoracic epidural, thoracic and upper abdominal surgery |
| Cappelleri *et al*, 2016(29) | Intraneural versus subparaneural injection in ultrasound-guided popliteal sciatic nerve block, orthopaedic surgery |
| Park *et al*, 2019(30) | Bupivacaine plus fentanyl versus bupivacaine alone in spinal anaesthesia with sedation, orthopaedic surgery |
| Teunkens *et al*, 2016(31) | Chloroprocaine versus bupivacaine versus lidocaine for spinal anaesthesia, orthopaedic surgery |
| Patnaik *et al*, 2018(32) | Landmark technique versus ultrasound guided paravertebral block, breast surgery |

**Supplementary Table S3: Randomised controlled trial papers used to develop sedation framework (n=12)**

| **Randomised controlled trial** | **Study details *(details used in purposive sampling, e.g. interventions compared, clinical area)*** |
| --- | --- |
| Bong *et al*, 2019(22) | Dexmedetomidine sedation with caudal block versus GA, paediatric surgery |
| Djaiani *et al*, 2016(33) | Dexmedetomidine versus propofol sedation, cardiothoracic surgery |
| Eberl *et al*, 2016(34) | Dexmedetomidine versus propofol sedation, endoscopy |
| Farag *et al*, 2017(35) | Dexmedetomidine versus propofol sedation, neurosurgery |
| Forster *et al*, 2018(36) | propofol sedation + lidocaine versus propofol alone, endoscopy |
| Goettel *et al*, 2016(37) | Dexmedetomidine versus propofol-remifentanil conscious sedation, neurosurgery |
| Mayr *et al*, 2016(7) | GA versus sedation, cardiothoracic surgery |
| Morue *et al*, 2018(38) | remifentanil + ketamine versus remifentanil alone, obstetrics |
| Schonenberger *et al*, 2016(39) | Conscious sedation versus GA, stroke |
| Sieber *et al*, 2018(40) and 2019(41) (+ Sieber, 2012 protocol(42) | Spinal anaesthesia plus deep versus light sedation, orthopaedic surgery |
| Silva-Jr *et al*, 2019(43) | Midazolam versus dexmedetomidine sedation, patients aged >70 years, clinical area not specified |
| Tan *et al*, 2016(44) | Dexmedetomidine versus midazolam sedation, urology surgery |

**References of randomised controlled trial papers used in Stage 1 of frameworks development (Tables S1-S3)**

1. Abrons RO, Zimmerman MB, El-Hattab YMS. Nasotracheal intubation over a bougie vs. non-bougie intubation: a prospective randomised, controlled trial in older children and adults using videolaryngoscopy. Anaesthesia. 2017;72(12):1491-500.

2. Basciani RM, Rindlisbacher A, Begert E, Brander L, Jakob SM, Etter R, et al. Anaesthetic induction with etomidate in cardiac surgery: A randomised controlled trial. Eur J Anaesthesiol. 2016;33(6):417-24.

3. Beck-Schimmer B, Bonvini JM, Braun J, Seeberger M, Neff TA, Risch TJ, et al. Which Anesthesia Regimen Is Best to Reduce Morbidity and Mortality in Lung Surgery?: A Multicenter Randomized Controlled Trial. Anesthesiology. 2016;125(2):313-21.

4. Dutta A, Sethi N, Sood J, Panday BC, Gupta M, Choudhary P, et al. The Effect of Dexmedetomidine on Propofol Requirements During Anesthesia Administered by Bispectral Index-Guided Closed-Loop Anesthesia Delivery System: A Randomized Controlled Study. Anesth Analg. 2019;129(1):84-91.

5. Ammar A, Mahmoud K, Elkersh A, Kasemy Z. A randomised controlled trial comparing the effects of propofol with isoflurane in patients with diastolic dysfunction undergoing coronary artery bypass graft surgery. Anaesthesia. 2016;71(11):1308-16.

6. Gebhardt V, Zawierucha V, Schöffski O, Schwarz A, Weiss C, Schmittner MD. Spinal anaesthesia with chloroprocaine 1% versus total intravenous anaesthesia for outpatient knee arthroscopy: A randomised controlled trial. Eur J Anaesthesiol. 2018;35(10):774-81.

7. Mayr NP, Hapfelmeier A, Martin K, Kurz A, van der Starre P, Babik B, et al. Comparison of sedation and general anaesthesia for transcatheter aortic valve implantation on cerebral oxygen saturation and neurocognitive outcome†. Br J Anaesth. 2016;116(1):90-9.

8. Mu J, Jiang T, Xu XB, Yuen VM, Irwin MG. Comparison of target-controlled infusion and manual infusion for propofol anaesthesia in children. Br J Anaesth. 2018;120(5):1049-55.

9. Oh CS, Lee J, Yoon TG, Seo EH, Park HJ, Piao L, et al. Effect of Equipotent Doses of Propofol versus Sevoflurane Anesthesia on Regulatory T Cells after Breast Cancer Surgery. Anesthesiology. 2018;129(5):921-31.

10. Ryu KH, Song K, Lim TY, Choi WJ, Kim YH, Kim HS. Does Equi-Minimum Alveolar Concentration Value Ensure Equivalent Analgesic or Hypnotic Potency?: A Comparison between Desflurane and Sevoflurane. Anesthesiology. 2018;128(6):1092-8.

11. Koo BW, Oh AY, Na HS, Lee HJ, Kang SB, Kim DW, et al. Effects of depth of neuromuscular block on surgical conditions during laparoscopic colorectal surgery: a randomised controlled trial. Anaesthesia. 2018;73(9):1090-6.

12. YaDeau JT, Fields KG, Kahn RL, LaSala VR, Ellis SJ, Levine DS, et al. Readiness for Discharge After Foot and Ankle Surgery Using Peripheral Nerve Blocks: A Randomized Controlled Trial Comparing Spinal and General Anesthesia as Supplements to Nerve Blocks. Anesth Analg. 2018;127(3):759-66.

13. YaDeau JT. Spinal versus General Anesthesia with Popliteal and Adductor Canal Blocks for Ambulatory Foot and Ankle Surgery. A Double-Blinded Randomized Controlled Trial. (Study Protocol, July 9^th^, 2018). Available from: https://classic.clinicaltrials.gov/ProvidedDocs/91/NCT02996591/Prot_SAP_001.pdf

14. Zhang Y, Shan GJ, Zhang YX, Cao SJ, Zhu SN, Li HJ, et al. Propofol compared with sevoflurane general anaesthesia is associated with decreased delayed neurocognitive recovery in older adults. Br J Anaesth. 2018;121(3):595-604.

15. Zhang Y, Li HJ, Wang DX, Jia HQ, Sun XD, Pan LH, et al. Impact of inhalational versus intravenous anaesthesia on early delirium and long-term survival in elderly patients after cancer surgery: study protocol of a multicentre, open-label, and randomised controlled trial. BMJ Open. 2017;7(11):e018607.

16. Choi SN, Jang YE, Lee JH, Kim EH, Kim JT, Kim HS. Comparison of rocuronium requirement in children with continuous infusion versus intermittent bolus: A randomised controlled trial. Eur J Anaesthesiol. 2019;36(3):194-9.

17. Fuchs-Buder T, Schmartz D, Baumann C, Hilt L, Nomine-Criqui C, Meistelman C, et al. Deep neuromuscular blockade improves surgical conditions during gastric bypass surgery for morbid obesity: A randomised controlled trial. Eur J Anaesthesiol. 2019;36(7):486-93.

18. Aitken E, Jackson A, Kearns R, Steven M, Kinsella J, Clancy M, et al. Effect of regional versus local anaesthesia on outcome after arteriovenous fistula creation: a randomised controlled trial. Lancet. 2016;388(10049):1067-74.

19. Macfarlane AJ, Kearns RJ, Aitken E, Kinsella J, Clancy MJ. Does regional compared to local anaesthesia influence outcome after arteriovenous fistula creation? Trials. 2013;14:263.

20. Arnuntasupakul V, Chalachewa T, Leurcharusmee P, Tiyaprasertkul W, Finlayson RJ, Tran DQ. Ultrasound with neurostimulation compared with ultrasound guidance alone for lumbar plexus block: A randomised single blinded equivalence trial. Eur J Anaesthesiol. 2018;35(3):224-30.

21. Boivin A, Nadeau MJ, Dion N, Lévesque S, Nicole PC, Turgeon AF. Ultrasound-Guided Single-Injection Infraclavicular Block Versus Ultrasound-Guided Double-Injection Axillary Block: A Noninferiority Randomized Controlled Trial. Anesth Analg. 2016;122(1):273-8.

22. Bong CL, Tan J, Lim S, Low Y, Sim SW, Rajadurai VS, et al. Randomised controlled trial of dexmedetomidine sedation vs general anaesthesia for inguinal hernia surgery on perioperative outcomes in infants. Br J Anaesth. 2019;122(5):662-70.

23. Grape S, Pawa A, Weber E, Albrecht E. Retroclavicular vs supraclavicular brachial plexus block for distal upper limb surgery: a randomised, controlled, single-blinded trial. Br J Anaesth. 2019;122(4):518-24.

24. Chin A, Crooke B, Heywood L, Brijball R, Pelecanos AM, Abeypala W. A randomised controlled trial comparing needle movements during combined spinal-epidural anaesthesia with and without ultrasound assistance. Anaesthesia. 2018;73(4):466-73.

25. Li M, Ni X, Xu Z, Shen F, Song Y, Li Q, et al. Ultrasound-Assisted Technology Versus the Conventional Landmark Location Method in Spinal Anesthesia for Cesarean Delivery in Obese Parturients: A Randomized Controlled Trial. Anesth Analg. 2019;129(1):155-61.

26. Ryu JH, Lee CS, Kim YC, Lee SC, Shankar H, Moon JY. Ultrasound-Assisted Versus Fluoroscopic-Guided Lumbar Sympathetic Ganglion Block: A Prospective and Randomized Study. Anesth Analg. 2018;126(4):1362-8.

27. Echaniz G, De Miguel M, Merritt G, Sierra P, Bora P, Borah N, et al. Bilateral suprazygomatic maxillary nerve blocks vs. infraorbital and palatine nerve blocks in cleft lip and palate repair: A double-blind, randomised study. Eur J Anaesthesiol. 2019;36(1):40-7.

28. Auyong DB, Hostetter L, Yuan SC, Slee AE, Hanson NA. Evaluation of Ultrasound-Assisted Thoracic Epidural Placement in Patients Undergoing Upper Abdominal and Thoracic Surgery: A Randomized, Double-Blind Study. Reg Anesth Pain Med. 2017;42(2):204-9.

29. Cappelleri G, Cedrati VL, Fedele LL, Gemma M, Camici L, Loiero M, et al. Effects of the Intraneural and Subparaneural Ultrasound-Guided Popliteal Sciatic Nerve Block: A Prospective, Randomized, Double-Blind Clinical and Electrophysiological Comparison. Reg Anesth Pain Med. 2016;41(4):430-7.

30. Park SK, Lee JH, Yoo S, Kim WH, Lim YJ, Bahk JH, et al. Comparison of bupivacaine plus intrathecal fentanyl and bupivacaine alone for spinal anesthesia with intravenous dexmedetomidine sedation: a randomized, double-blind, noninferiority trial. Reg Anesth Pain Med. 2019;44(4):459-65.

31. Teunkens A, Vermeulen K, Van Gerven E, Fieuws S, Van de Velde M, Rex S. Comparison of 2-Chloroprocaine, Bupivacaine, and Lidocaine for Spinal Anesthesia in Patients Undergoing Knee Arthroscopy in an Outpatient Setting: A Double-Blind Randomized Controlled Trial. Reg Anesth Pain Med. 2016;41(5):576-83.

32. Patnaik R, Chhabra A, Subramaniam R, Arora MK, Goswami D, Srivastava A, et al. Comparison of Paravertebral Block by Anatomic Landmark Technique to Ultrasound-Guided Paravertebral Block for Breast Surgery Anesthesia: A Randomized Controlled Trial. Reg Anesth Pain Med. 2018;43(4):385-90.

33. Djaiani G, Silverton N, Fedorko L, Carroll J, Styra R, Rao V, et al. Dexmedetomidine versus Propofol Sedation Reduces Delirium after Cardiac Surgery: A Randomized Controlled Trial. Anesthesiology. 2016;124(2):362-8.

34. Eberl S, Preckel B, Bergman JJ, van Dieren S, Hollmann MW. Satisfaction and safety using dexmedetomidine or propofol sedation during endoscopic oesophageal procedures: A randomised controlled trial. Eur J Anaesthesiol. 2016;33(9):631-7.

35. Farag E, Kot M, Podolyak A, Argalious M, Deogaonkar M, Mascha EJ, et al. The relative effects of dexmedetomidine and propofol on cerebral blood flow velocity and regional brain oxygenation: A randomised noninferiority trial. Eur J Anaesthesiol. 2017;34(11):732-9.

36. Forster C, Vanhaudenhuyse A, Gast P, Louis E, Hick G, Brichant JF, et al. Intravenous infusion of lidocaine significantly reduces propofol dose for colonoscopy: a randomised placebo-controlled study. Br J Anaesth. 2018;121(5):1059-64.

37. Goettel N, Bharadwaj S, Venkatraghavan L, Mehta J, Bernstein M, Manninen PH. Dexmedetomidine vs propofol-remifentanil conscious sedation for awake craniotomy: a prospective randomized controlled trial. Br J Anaesth. 2016;116(6):811-21.

38. Morue HI, Raj-Lawrence S, Saxena S, Delbaere A, Engelman E, Barvais LA. Placebo versus low-dose ketamine infusion in addition to remifentanil target-controlled infusion for conscious sedation during oocyte retrieval: A double-blinded, randomised controlled trial. Eur J Anaesthesiol. 2018;35(9):667-74.

39. Schönenberger S, Uhlmann L, Hacke W, Schieber S, Mundiyanapurath S, Purrucker JC, et al. Effect of Conscious Sedation vs General Anesthesia on Early Neurological Improvement Among Patients With Ischemic Stroke Undergoing Endovascular Thrombectomy: A Randomized Clinical Trial. JAMA. 2016;316(19):1986-96.

40. Sieber FE, Neufeld KJ, Gottschalk A, Bigelow GE, Oh ES, Rosenberg PB, et al. Effect of Depth of Sedation in Older Patients Undergoing Hip Fracture Repair on Postoperative Delirium: The STRIDE Randomized Clinical Trial. JAMA Surgery. 2018;153(11):987-95.

41. Sieber F, Neufeld KJ, Gottschalk A, Bigelow GE, Oh ES, Rosenberg PB, et al. Depth of sedation as an interventional target to reduce postoperative delirium: mortality and functional outcomes of the Strategy to Reduce the Incidence of Postoperative Delirium in Elderly Patients randomised clinical trial. Br J Anaesth. 2019;122(4):480-9.

42. Sieber FE. A Strategy to Reduce the Incidence of Post‐Operative Delirium in Elderly patients (The STRIDE Study) (Study Protocol, November 19^th^, 2012). Available from: https://classic.clinicaltrials.gov/ProvidedDocs/07/NCT00590707/Prot_SAP_000.pdf

43. Silva-Jr JM, Katayama HT, Nogueira FAM, Moura TB, Alves TL, de Oliveira BW. Comparison of dexmedetomidine and benzodiazepine for intraoperative sedation in elderly patients: a randomized clinical trial. Reg Anesth Pain Med. 2019;44(3):319-24.

44. Tan WF, Miao EY, Jin F, Ma H, Lu HW. Changes in First Postoperative Night Bispectral Index After Daytime Sedation Induced by Dexmedetomidine or Midazolam Under Regional Anesthesia: A Randomized Controlled Trial. Reg Anesth Pain Med. 2016;41(3):380-6.
