# Supplementary material for: Frameworks for the design and reporting of anaesthesia interventions in perioperative clinical trials
Source: BJA Open. 2025 Feb 4;13:100374. doi: 10.1016/j.bjao.2024.100374 (PMC11847521; doi:10.1016/j.bjao.2024.100374)
Supplement: Multimedia component 4 [file mmc4.docx]

**Supplementary Appendix S4: Summary of key changes made to anaesthetic frameworks following focus groups and interviews**

| **All frameworks** |
| --- |
| Ensuring in the introductory information the importance of using these frameworks at trial design stage |
| The word ‘typology’ changed to ‘framework’ for clarity |
| Definitions for each mode of anaesthesia added with references |
| Terminology ‘Person delivering GA/RA/sedation’ used in place of ‘Expertise’ or ‘Operator’ to improve clarity |
| Allow for study team to define required expertise in study intervention rather than frameworks specifying levels of expertise |
| Importance of ensuring it’s clear that Setting refers to the setting of where anaesthetic intervention delivered |
| Consistency of Setting sub-categories across all three frameworks to include hospital and non-hospital locations and improve clarity of wording. (e.g. ‘Remote location’ not well-understood). |
| Consistency throughout all three frameworks of details of drugs used including dosing |
| Importance of specifying the sub-categories within ‘Standard’ monitoring as these may not be standard everywhere and ensure they are the same across all three frameworks |
| Define ‘recovery’ as meaning until discharge back to usual residence |
| Include both ‘PACU’ and ‘recovery area’ in post-procedure destinations as can mean different things |
|  |
| **General anaesthesia framework** |
|  |
| Clarify that pre-procedural medications are over and above patient’s usual medications (also for sedation) |
| Use of a timeline approach for drugs given during GA |
|  |
| **Regional anaesthesia framework** |
|  |
| The importance of broadening the definition and wording used for the RA framework so that it could encompass regional techniques to deliver other types of medications (not just anaesthestic agents). |
| ‘Type and location of regional procedure used’ section expanded, re-structured and re-worded to be more comprehensive and clear |
| ‘Block room’ added as an additional in-hospital setting for RA framework specifically |
| Importance of adding in consent and correct side of body to block |
| Specification of sub-categories on how to describe injection |
| Re-wording to ‘Escalation of care if required’ instead of ‘Alternative escalation’ |
|  |
| **Sedation framework** |
| Clarify that pre-procedural medications are over and above patient’s usual medications (also for GA) |
| Restructuring of sections into ‘sedation drug(s) used’ and ‘parameters used for sedation titration’ to flow more logically. Allow for bolus or infusion delivery of sedation drug (with target controlled and non-target controlled infusion options). Addition of ‘other modes of sedation’ to be more comprehensive. |
| Clarification that additional techniques section relates to ‘Unplanned additional techniques’ |
